# High Frequency Jet Ventilation during stereotactic ablation of liver tumours: an observational study on blood gas analysis as a measure of lung function during general anaesthesia

**DOI:** 10.12688/f1000research.18369.1

**Published:** 2019-04-05

**Authors:** Karolina Galmén, Jan G Jakobsson, Jacob Freedman, Piotr Harbut

**Affiliations:** 1 Department of Anaesthesia and Intensive Care, Institution for Clinical Sciences, Karolinska Institutet, Danderyd University Hospital, Stockholm, 182 88, Sweden; 2 Department of Surgery, Institution for Clinical Sciences, Karolinska Institutet, Danderyd University Hospital, Stockholm, 182 88, Sweden

**Keywords:** High-Frequency Jet Ventilation, Blood Gas Analysis, Anesthesia, General, Liver Neoplasms, Stereotaxic Techniques, Surgery, Computer-Assisted/methods

## Abstract

**Background:** Stereotactic ablation of tumours in solid organs is a promising curative procedure in clinical oncology. The technique demands minimal target organ movements to optimise tumour destruction and prevent injury to surrounding tissues. High frequency jet ventilation (HFJV) is a novel option during these procedures, reducing the respiratory-associated movements of the liver. The effects of HFJV via endotracheal catheter on gas exchange during liver tumour ablation is not well studied.

**Methods:** The aim of this explorative study was to assess lung function and the effects on blood gas and lactate during HFJV in patients undergoing stereotactic liver ablation. Blood gases were analysed in 25 patients scheduled for stereotactic liver ablation under general anaesthesia pre-induction, every 15 minutes during HFJV and following extubation in the recovery room. The HFJV was set at fixed settings.

**Results:** None of the patients developed hypoxia or signs of increased lactate production but a great variation in PaO _2_/FiO _2_ ratio was found; from 13.1 to 71.3. An increase in mean PaCO _2_ was observed, from a baseline of 5.0 to a peak of 7.1 at 30 minutes (p <0.001) and a decrease was found in median pH, from a baseline of 7.44 to 7.31 at 15 minutes (p=0.03). We could not see any clear association between a decrease in PaO _2_/FiO _2_ ratio and PaCO _2_ elevation.

**Conclusions:** HFJV during general anaesthesia in patients undergoing stereotactic liver ablation is feasible and it did not cause hypoxemia or signs of increased lactate production. A reversible mild to moderate impairment of gas exchange was found during HFJV.

## Introduction

Stereotactic ablation of tumours in solid organs is a promising, dynamically developing and potentially curative procedure in clinical oncology. High precision in targeting malignant lesions with the use of image fusion for pre-interventional diagnostic imaging is highly dependent on immobilisation of the operation field. Respiratory movements cause diaphragmatic shifts, displacing abdominal organs during stereotactic-guided procedures. Biro *et al.* ^1^ observed that breathing-related liver motion decreased from 20mm to 5mm using high frequency jet ventilation (HFJV) as compared to conventional ventilation.

HFJV is an attractive novel ventilatory strategy, minimizing movements in proximity to the lungs and keeping the abdominal and extraperitoneal target organ immobilised ^2^. The surgical ablation procedure usually requires at least 30 to 45 minutes to be performed and as the patient is anesthetised before ablation begins and a CT-scan is performed before the procedure can start, HFJV will thus be applied for up to at least an hour not uncommonly in elderly ASA Class 2-3 patients (ASA Class: American Association of Anesthesiologists Classification, ASA 2 being a patient with mild systemic disease and ASA 3 being a patient with severe systemic disease). The effects on oxygenation, carbon dioxide elimination, pH and lactate formation are not well-studied in this patient category during HFJV with the jet catheter placed through the endotracheal tube during liver ablation.

The primary aim of this explorative observational study was to study changes over time in arterial oxygenation, carbon dioxide elimination, pH and lactate formation in adult patients undergoing liver ablation under general anaesthesia who are ventilated with HFJV. The secondary aim was to describe the PaO _2_/FiO _2_ ratio and its relation to PaCO _2_ during HFJV.

## Methods

### Ethics

This study conforms to the standard of the Declaration of Helsinki and the study was approved by the Regional Ethics Committee in Stockholm (Dnr 2016/1124-32, June 7 ^th^, 2016) and the local Radiation Protection Committee at Danderyd University Hospital (Project number 2016-1, June 1 ^st^, 2016). Written informed consent for participation and publication was obtained from all subjects.

### Patient population

Twenty-five consecutive patients (age>=50 years) with primary or secondary malignant liver tumours accepted for elective stereotactic ablation were included in this study after obtaining their written and oral informed consent. Exclusion criteria applied before inclusion of the 25 patients were pregnancy, recent pneumothorax or severe, poorly controlled lung disease. Patient demographics are presented in Table 1. The study was conducted October-December 2017 at Danderyd University Hospital, Stockholm, Sweden. ****

**Table 1.** Demographics. Study population demographics.

Variables		Overall series
Age (years)	Median Mean	70 68 (9.649 SD)
Gender, n (%)	Male Female	17 (68) 8 (32)
BMI (kg/m ^2^)	Mean	26.7
ASA-score	ASA 1 ASA 2 ASA 3	1 * 6 18
Smoking	Yes No	3 (12) 22 (88) **
Lung disease	Yes No	6 (24) *** 19 (76)
Time in HFJV	Median (min)	75

* number of patients in each ASA-classification group ** 8 patients with previous smoking habits ***2 patients with mild asthma, 2 patients with lung metastasis, 1 patient with pulmonary hypertension and Sjogren’s disease, 1 patient with earlier postoperative pulmonary embolism.

### Anaesthetic methods

All patients had total intravenous anaesthesia based on propofol (Propofol-Lipuro®, B. Braun Melsungen AG, Melsungen, Germany) 3–12 mg/kg/min and remifentanil (Ultiva®, GlaxoSmithKline AB, Solna, Sweden) 0,05–2 mikrgr/kg/min for induction and maintenance. Neuromuscular block was achieved by IV injection of rocuronium (Esmeron®, MSD, Haarlem, Netherlands) 0,6 mg/kg at induction. **

The patients were preoxygenated with FiO _2_ of 0.8 at induction and intubated with an endotracheal tube (ETT) one size larger than the standard (i.e. size 8 for women and size 9 for men) to create sufficient space for the jet cannula and allow passive exhalation. An alveolar recruitment maneuver was performed following endotracheal intubation and confirmation of correct ETT placement and, subsequently, initiation of conventional ventilation was carried out while preparing for the HFJV.

The HFJV was performed with a Monsson III device ( *Acutronic, Switzerland*). A jet cannula ( *Laserjet 40, double lumen jet catheter acc Biro, Acutronic Medical System AG, Hirzel, Germany*) was put through the ETT. The jet ventilator was initiated with standardised settings on the jet ventilator; driving pressure (DP) between 1.2-1.4 bar, frequency of 220 min ^-1^, oxygen 80% throughout the procedure. The ventilator had pre-set limits for ventilatory pressures at which ventilation is aborted and an alarm sound is activated.

The following protocol for a raise in PaCO _2_ was used. When PaCO _2_ rises above 10 kPa, DP is increased. If this does not lead to an acceptable level of PaCO _2_, the frequency is decreased. If still not a satisfactory PaCO _2_, HFJV is aborted and conventional ventilation is started. All these steps are carried out in close communication with the surgeon.

Monitoring consisted of 3-lead ECG, oxygen saturation via pulse oximetry on a finger, invasive blood pressure via an arterial line in radial artery and level of muscle relaxation through train of four (TOF), recorded every 5 minutes in accordance with routine practice.

### Blood gas analysis

All patients had an arterial line inserted in accordance with routine practice for patients having HFJV. In 19 patients it was placed before anaesthesia and in 6 patients right after induction. Blood gas and lactate were sampled at baseline, after the start of HFJV, every 15 minutes (15’, 30’ and 45’) during the procedure and when extubated in the recovery room. No further follow-up was done; the study protocol ended with the postoperative blood gas analysis.

Blood gases were analysed with a standard ABL 90 FLEX ( *Radiometer Medical Aps, Brönshöj, Denmark*) in a standard blood gas syringe and was analysed immediately after the blood was taken from the patient (approximately 1–3 minutes).

PaCO _2_ was categorised in three groups; <6 kPa, 6-8 kPa and >8 kPa. PaO _2_/FiO _2_ ratio was categorised in three groups; <20, 20-40 and >40.

### Statistics

This is an observational study thus; no power analysis or statistical plan has been conducted and we planned for 25 patients to target the primary outcomes. Data is presented as mean and SD for normal distributed data and median and range for non-normal distributed data. Data was tested for normality with the Shapiro-Wilk test. Repeated measures one-way ANOVA was used on normally distributed data and repeated measures ANOVA on ranks was used when data was non-normally distributed. The Bonferroni or Tukey Test was used in all pairwise multiple comparison procedures. Missing data; calculations are made on available data only. A p<0.05 was considered statistically significant. All statistical tests were conducted with SigmaPlot ( *version 14, Software Inc., San Jose, California, USA*).

## Results

All 25 patients had an uncomplicated perioperative course. Anaesthesia, surgery and early recovery was uneventful apart from one patient who experienced a minor pneumothorax associated with the surgical procedure and was left with no further intervention. Changes in the HFJV settings per the protocol for increasing PaCO _2_ had to be initiated in one patient. HFJV did not need to be discontinued in any patients. One patient had a DP of 1,6 during the whole procedure. In one patient, oxygen was raised from 80% to 100% after 5 min of HFJV as saturation dropped to 93%. Saturation was then normalised.

A total number of 131 blood gases were analysed in this study.

**Figure 1.**
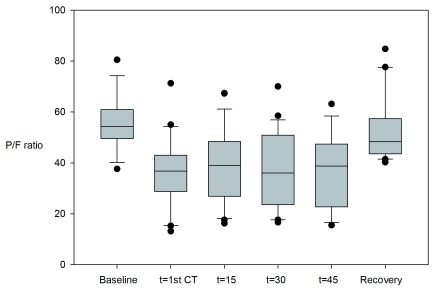
PaO _2_/FiO _2_ ratio. PaO _2_/FiO _2_ ratio at baseline, during high frequency jet ventilation and after extubation in the recovery room. An inter-individual variation was seen in oxygenation during HFJV and the PaO _2_/FiO _2_ ratio significantly decreased but was restored at recovery.

Mean PaO _2_ at baseline was 11.6 kPa (SD 2.3). All patients had a PaO _2_ above the normal lower limit of 8 kPa except for one patient (7.9 kPa). The PaO _2_ increased during HFJV and all patients had perioperative PaO _2_>10 kPa (see Table 2). An inter-individual variation was seen in oxygenation during HFJV and the PaO _2_/FiO _2_ ratio significantly decreased but was restored at recovery (see Figure 1). During the stay in the recovery room, three patients required supplementary oxygen and in two cases values were not accessible. All remaining 20 patients breathing room air had a PaO _2_ above the normal lower limit of 8 kPa (see Table 2).

**Table 2.** PaO _2_. PaO _2_ at baseline, during high frequency jet ventilation (HFJV) and after extubation in the recovery room. No hypoxemia was seen during HFJV. At recovery 3 patients required additional oxygen.

Group	N	Missing	Mean	SD	Range
Baseline	25	6	11.6	2.3	7.9–16.9
at t=1 ^st^ CT	25	1	29.2	10.7	10.5–57
at t=15’ later	25	1	30.7	11.4	13–53.9
at t=30’ later	25	3	29.7	12.4	13.3–56
at t=45’ later	25	6	29.8	11.8	12.4–50.5
Recovery	25	5	11.4	3.0	8.45–17.8

**Figure 2.**
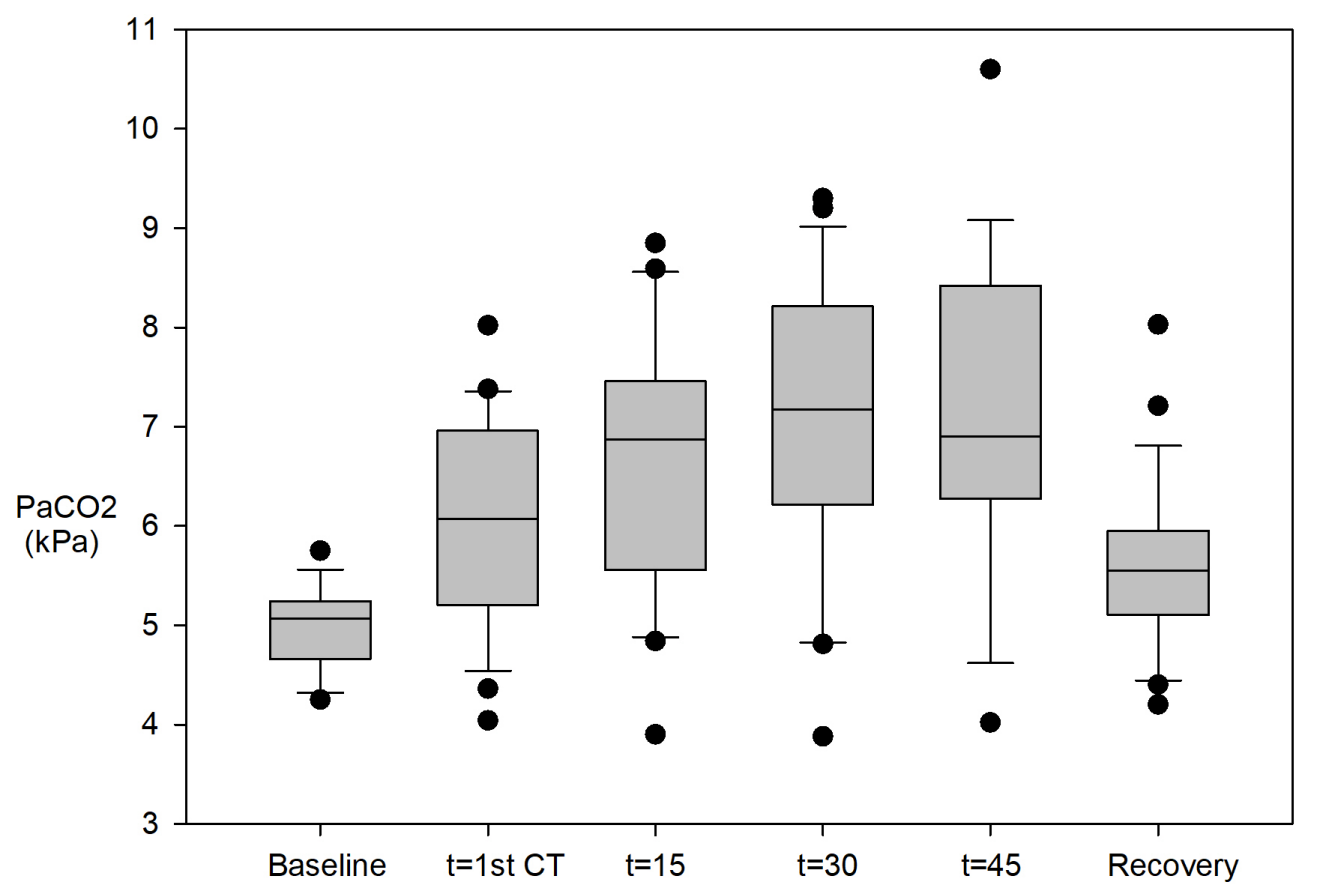
PaCO _2_. PaCO _2_ at baseline, during high frequency jet ventilation and after extubation in the recovery room. A significant raise was seen in mean PaCO _2_ from baseline to 1 ^st^ CT. Five out of 23 patients had a PaCO _2_ value >6 kPa at recovery, the highest value being 8.03 kPa.

Mean PaCO _2_ at baseline was 5.0 (SD 0.4). None of the patients had a PaCO _2_ above the normal limit of 6 kPa. A significant raise was seen in mean PaCO _2_ from baseline to t=1 ^st^ CT; 5.0 to 6.1 kPa (p=0.003). The mean PaCO _2_ value increased further to peak mean PaCO _2_ 7.1 kPa at 45’ (see Figure 2). The number of patients with a PaCO _2_ >6 kPa was 14/24 at t=1 ^st^ CT, 15/24 at t=15’, 18/22 at t=30’, 15/19 patients at t=45’. Mean PaCO _2_ during recovery (5,6 kPa) was not significantly different from the mean baseline. Five out of 23 patients had a PaCO _2_ value >6 kPa at recovery, the highest value being 8.03 kPa.

**Figure 3.**
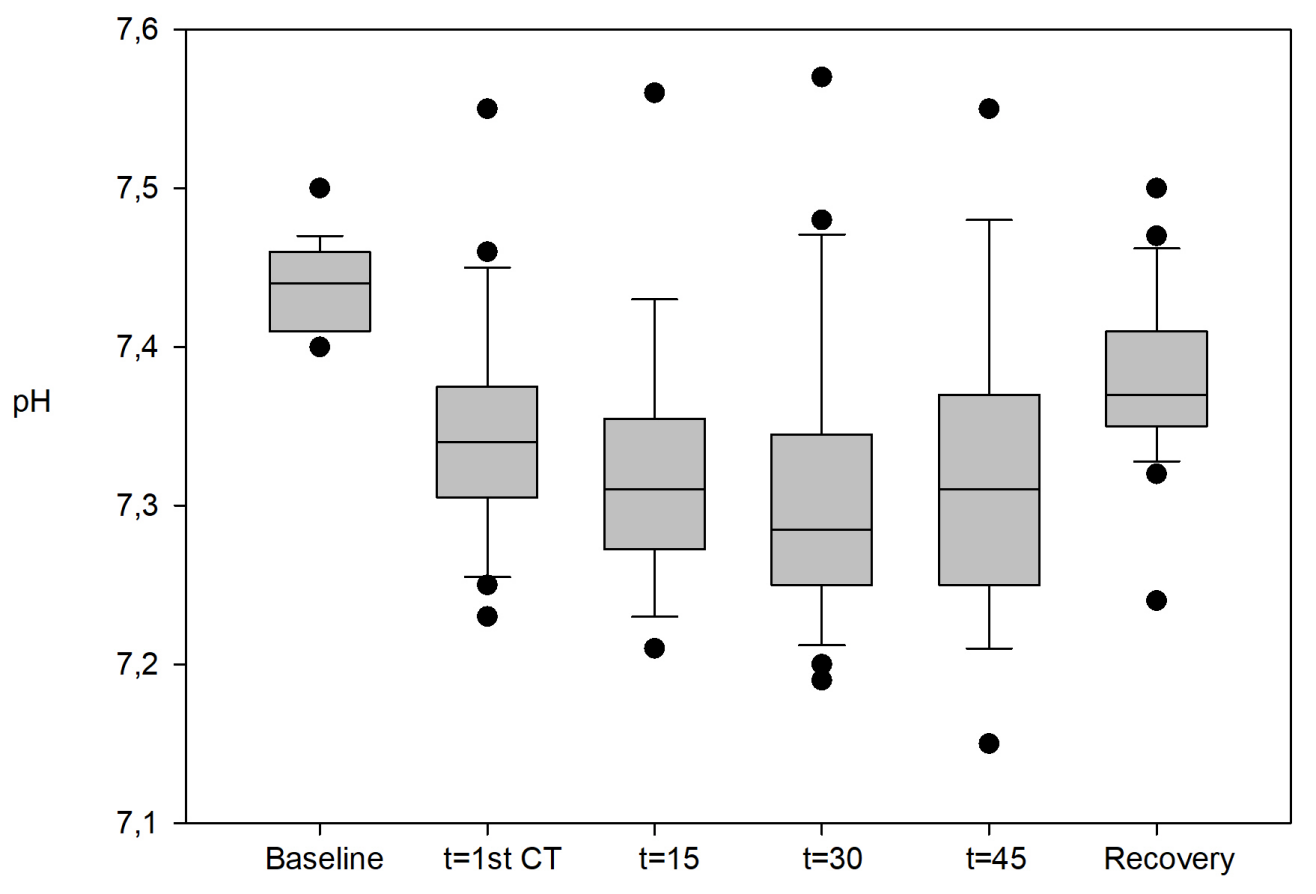
pH. pH at baseline, during high frequency jet ventilation and after extubation in the recovery room. There was a significant drop in pH from baseline during HFJV. Four out of 23 patients had a pH<7.35 at recovery, with the lowest value being 7.24.

Median pH decreased from baseline 7.44 to 7.31 at t=15’ (p=0.03). There was a small further drop in pH but with no statistical difference between time points (see Figure 3). Fourteen out of 24 patients had a pH <7.35 at t=1 ^st^ CT, 18/24 patients at t =15’, 17/22 at t =30’ and 14/19 at t=45’. Median pH during recovery (7.37) was not significantly different from the median baseline. Four out of 23 patients had a pH<7.35 at recovery, with the lowest value being 7.24 (see Figure 3).

All lactate values were within normal range during HFJV. One patient had a minor increase (2.3 mmol L ^-1^) during recovery.

**Figure 4.**
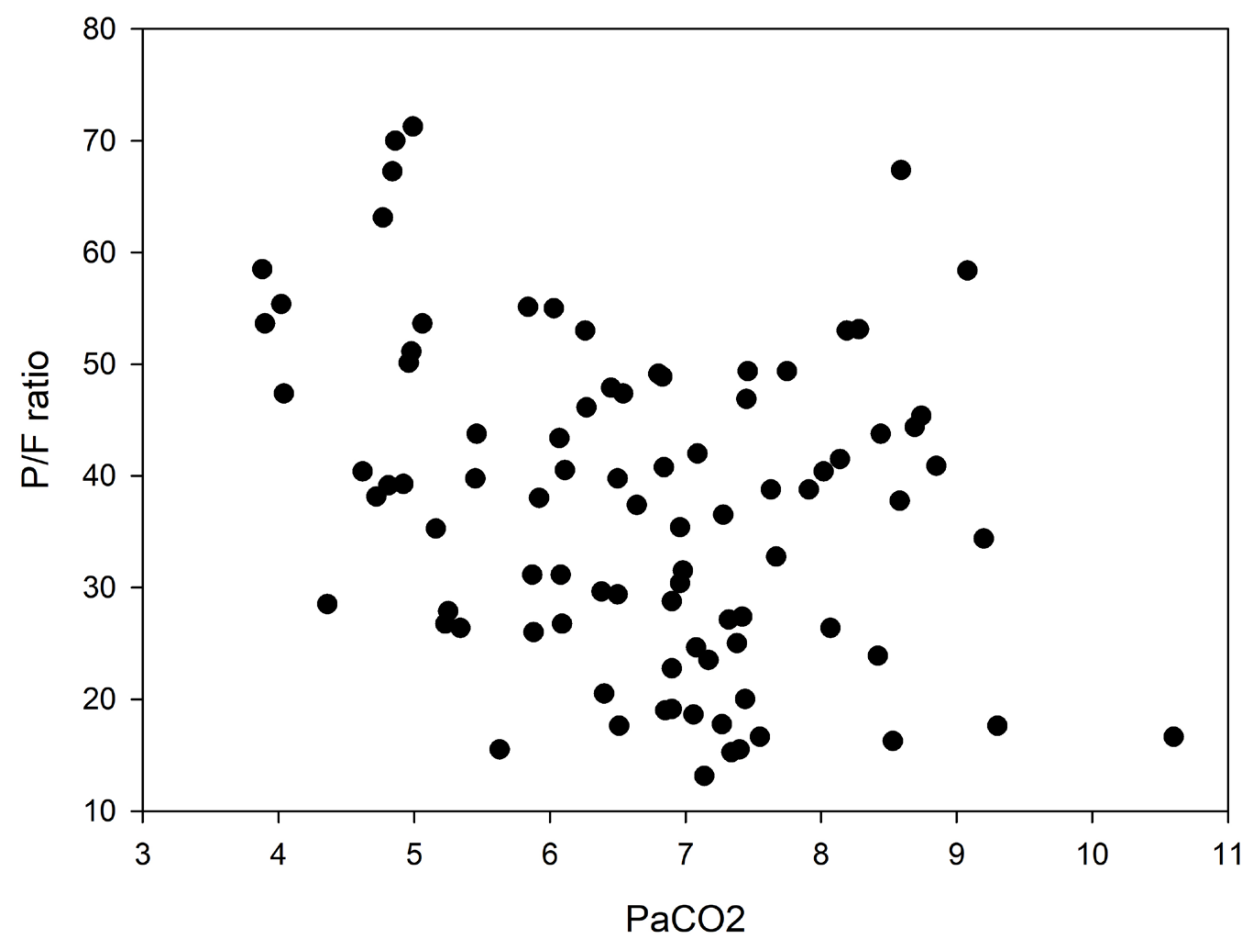
PaO _2_/FiO _2_ ration vs PaCO _2_. Plotted blood gas pairs of PaO _2_/FiO _2_ ration vs PaCO _2_ in the 25 patients studied. No clear correlation could be seen.

There was no clear correlation between decrease in PaO _2_/FiO _2_ ration and the PaCO _2_ increase (see Figure 4). Ten out of 16 blood gas analyses (62%) with a PaCO _2_>8 had a PaO _2_/FiO _2_ ratio of >40. Thirteen out of 39 (33%) of the blood gas analyses with normal PaCO _2_ had PaO _2_/FiO _2_ ratio <40.

## Discussion

There is sparse information about arterial blood gas changes during HFJV during abdominal surgery. No previous study has explicitly assessed oxygenation and carbon dioxide elimination during CT-guided stereotactic liver ablation with the jet ventilation catheter placed inside an ETT. Our study showed that this technique, high frequency jet ventilation by a catheter placed through the ETT, maintained adequate oxygenation in all patients. The gas exchange, the ratio of arterial oxygen partial pressure to fractional inspired oxygen (PaO _2_/FiO _2_), decreased and varied considerably. The carbon dioxide tension increased and pH decreased reasonably as an effect of the CO _2_ retention. Furthermore, the impairment in oxygenation, decrease in PaO _2_/FiO _2_ ratio and CO _2_ elimination was found somewhat surprisingly not to correlate, showing a scattered pattern. There was also only modest residual impairment of gas exchange during early recovery. ****

High frequency ventilation is not new ^3^. However, most HFJV use is for airway interventions, for example during a bronchoscopy or laryngoscopy. This may not entirely translate to its use in abdominal surgery, in this case during puncture of the liver under CT-guidance, with the jet catheter placed through an endotracheal in patients who are under general anaesthesia and receiving muscle relaxants. The HFJV catheter in our study was inserted into the endotracheal tube and thus the explicit effective inspired oxygen fraction cannot be defined. The PaO _2_/FiO _2_ findings may thus not be entirely true. The open system may also impact the expired pressure as well as gas elimination. Pause pressure was measured via the tip of the jet cannula, providing the pressure at tracheal level between the jet inspirations. This is not a fully representative pressure for the whole lung but may still be seen as an indicator for built-up auto positive end expiratory pressure (PEEP). The HFJV technique is dependent on passive outflow, contrary to high frequency oscillation that possibly has a more active exhalation. There is indeed, with HFJV, an obvious risk of carbon dioxide retention as well as a potential for a build-up of intrinsic PEEP and subsequent risk for barotrauma.

All patients had total intravenous anaesthesia because of the open airway system and all had muscle relaxation to promote the stereotactic liver ablation, both factors that impact on lung function ^4^. Age and supine position are also factors known to impair the lung function during anaesthesia ^5^. In the present study a FiO _2_ of 0.8 was used during the entire procedure except for one patient who had a FiO _2_ of 1.0. High oxygen fraction may cause rapid absorption atelectasis and anaesthesia *per se* causes loss of muscle tone and reduced lung volume, factors having a negative effect on ventilation/perfusion matching ^6^. It should also be acknowledged that preoxygenation was performed before induction, thus further promoting lung collapse when the high oxygen fraction is maintained ^7^. Several of the patients had a history of pulmonary disease, further impairing ventilation/perfusion matching during anaesthesia ^8^. The oxygenation as well as the arterial carbon dioxide tension was somewhat surprisingly restored by the arterial blood gas measurement in the recovery room. Lung recruitment manoeuvre is standard procedure following intubation but not during emergencies.

### Comparison with previous studies

Bickel *et al*. ^9^ found that HFJV during laparoscopy in ASA 1-2 patients lessened the cardiovascular effects of pneumoperitoneum as compared to conventional ventilation. They only briefly commented on the effects on blood gases. Contrary to the present findings, they state that PaO _2_, PaCO _2_ and pH was similar during all phases and between the study groups. Explicit values were, however, not presented. They used the same type of HFJV apparatus, with an average ventilator frequency of 150 cycles/min. In an earlier study, transtracheal HFJV was started with 100% oxygen at 30 to 35 pounds per square inch of driving pressure (equals 2.1-2.4 bar), 100 cycles per minute and an I:E ratio of 25%. An increase in CO _2_ was noted at 10 minutes, similar to the present findings ^10^. There is also a risk for hypercapnia and/or hypoxia when HFJV is used during airway surgery as shown by Fernandez-Bustamante *et al.* ^11^. Their study included 316 patients who underwent an interventional rigid bronchoscopy under general anaesthesia and HFJV. The most common complications were hypoxia, hypercapnia and hemodynamic instability. Sutterlin *et al.* ^12^ found that increasing frequency raised arterial carbon dioxide, possibly by reducing the gas elimination time. The carbon dioxide and pH normalised after awakening. It would indeed be of value to be able to monitor the inspired alveolar oxygen pressure and carbon dioxide tension as well as PEEP during HFJV.

### Alternative methods and future studies

Alternative lung ventilation modes could be considered during liver tumour ablation instead of HFJV. Various forms of high flow systems for apnoeic oxygenation have recently gained interest, including THRIVE (transnasal humidified rapid-insufflation ventilatory exchange). Several reports show the feasibility of THRIVE during general anaesthesia in the difficult airway situation ^13^ and in laryngeal surgery ^14^. In the difficult airway situation, THRIVE is a way of prolonging the apnoeic time until a definitive airway is secured. Whether this technique could have a place in anaesthetic management for stereotactic liver ablation needs to be investigated.

### Limitations of the study

There are several limitations that should be considered. We have not included any control group having conventional ventilation as this would jeopardise intervention safety and accuracy, increasing the risk for bleeding and tissue damage. As pointed out, only one HFJV setting was used. Pressure and frequency was not adjusted, except in one patient when pressure was slightly adjusted according to a raise in PaCO _2_. Likewise, a fixed oxygen fraction was used throughout the procedure and, as mentioned, oxygen concentration reaching the alveoli and PEEP levels cannot be assessed. The focus of our study was gas exchange during HFJV, following arterial blood gases under steady state jet ventilation. We did not include any further follow-up after the recovery phase.

## Conclusion

This study shows the feasibility of using HFJV through an ETT with an FiO _2_ of 0.8 during liver tumour ablation. It did not cause any hypoxia or increase in lactate. It was associated with mild to moderate impairment in gas exchange that was restored during early recovery.

## Data availability

### Underlying data

Harvard Dataverse: Replication Data for; High Frequency Jet Ventilation during stereotactic ablation of liver tumours – an observational study on blood gas analysis as a measure of lung function during general anaesthesia. https://doi.org/10.7910/DVN/MXHFWW ^15^

This project contains the following underlying data:

- Bloood gas HFJV F1000_data_190401.tab (Blood gas data associated to HFJV)

Data are available under the terms of the Creative Commons Zero "No rights reserved" data waiver (CC0 1.0 Public domain dedication).
