# Supplementary material for: High Frequency Jet Ventilation during stereotactic ablation of liver tumours: an observational study on blood gas analysis as a measure of lung function during general anaesthesia
Source: F1000Res. 2019 Apr 5;8:386. [Version 1] doi: 10.12688/f1000research.18369.1 (PMC6753604; doi:10.12688/f1000research.18369.1)
Supplement: Peer review history

# Peer review: Reviewer response for version 1

**DOI:** 10.5256/f1000research.20098.r46793

**Published:** 2019-09-23

**Authors:** Göran Hedenstierna

**Affiliations:** 1 Hedenstierna Laboratory, Department of Medical Sciences, Uppsala University Hospital, Uppsala, Sweden

HFJV causes minor movement of liver tissue and is thus advantageous in liver ablation, compared to standard mechanical ventilation. This study shows that gas exchange can be maintained at a reasonable level for an hour-long anethesia/surgery. A successive fall in PaO2 was seen but in no patient was hypoxemia seen. Similarly, PaCO2 rose during the hour-long observation period, but not to any critical level. The authors found no correlation between fall in PaO2 and increase in PaCO2. Thus, hypoventilation was not the major or only cause of impaired oxygenation. FIO2 was in most patients 0.8, but in an open system, as here, the alveolar FO2 may be different and also vary between patients. The authors discuss a certain degree of auto-PEEP but have not been able to make measurements of it. Atelectasis may be reduced with increase in auto-PEEP, as inferred by the authors. One may speculate in varying distance between catheter tip and carina with effects on ventilation of each lung. Impairment in lung perfusion, possibly reflected by decrease in cardiac output, might be an additional cause of varying oxygenation.

 However, all these comments are speculations and need not be in a paper.

 On the whole, the study is well performed in a simple and straightforward way. Results are clear and show a reasonably maintained gas exchange under the studied conditions.

 My only minor criticism is that the ventilatory technique is mentioned in different parts of the paper and could be brought together in the Methods section.

I confirm that I have read this submission and believe that I have an appropriate level of expertise to confirm that it is of an acceptable scientific standard.

# Peer review: Reviewer response for version 1

**DOI:** 10.5256/f1000research.20098.r46791

**Published:** 2019-09-18

**Authors:** Astrid Bergmann

**Affiliations:** 1 Department of Anesthesiology and Intensive Care Medicine, Otto von Guericke University Magdeburg, Magdeburg, Germany

The authors address the question if high frequency jet ventilation (HFJV) affects blood gases when applied during sterotactic ablation of liver tumours. This procedure has evolved as an auspicious treatment in the field of hepatic oncology. It is crucial to minimize the movement of the liver to establish optimal conditions for the surgeon throughout the intervention. Hence, HFJV has evolved as a promising method. No data are available so far on the effects of HFJV on gas exchange during stereotactic ablation of liver tumours. Therefore, 25 patients have been involved in this observational study. Blood gases were assessed before induction and every 15 minutes during HFJV and finally in the recovery area after extubation. No hypoxia could be observed in any of the patients even though *p*aO _2_/F _I_O _2_ showed a wide range. *P*aCO _2_ increased during HFJV and returned to baseline levels after extubation. pH decreased due to CO _2_ retention during HFJV. There was no correlation between *p*aO _2_/F _I_O _2_ and CO _2_. The authors conclude that HFJV does not cause hypoxaemia during stereotactic liver ablation under general anaesthesia but that gas exchange is slightly impaired during the procedure. 

 This article makes an important contribution to the anaesthetic practice during stereotactic ablation of liver tumours. Working conditions for the surgeon can be optimised by HFJV without compromising the patient's gas exchange and metabolic status.

 The manuscript is well written and attractive. The abstract fulfils its purpose in giving a clear overview of the whole study. The background of the intervention as well as the importance in the oncological field and the problems associated with ventilation are clearly pictured in the introduction. A sound description of the aims of the study are also provided. The study population, anaesthetic methods and blood gas analysis are soundly described in the methods section. The presentation of the results is clear and straightforward with well-designed graphs and tables to illustrate the outcome parameters. Every result is discussed and literature in the field is cited and compared with the findings of this study. A perspective for future studies is given and the promising conclusion is drawn that HFJV is feasible to use during liver tumour ablation without causing hypoxia or an increase in lactate although a moderate impairment in gas exchange was observed during the procedure.

 All in all, I think that this paper is an important addition to the literature with a well-thought-out protocol, clear results and a promising conclusion. It is scientifically sound and should be accepted for indexing.

I confirm that I have read this submission and believe that I have an appropriate level of expertise to confirm that it is of an acceptable scientific standard.

# Peer review: Reviewer response for version 1

**DOI:** 10.5256/f1000research.20098.r49490

**Published:** 2019-07-03

**Authors:** Marzenna Podhorska-Okolow

**Affiliations:** 1 Department of Human Morphology and Embryology, Wroclaw Medical University, Wrocław, Poland; 2 Divison of Ultrastructure Research, Wroclaw Medical University, Wrocław, Poland

High frequency jet ventilation (HFJV) is an alternative method to conventional ventilation which is known to minimize movement of the thorax and abdomen what finally could facilitate surgical procedures. The aim of the study of the revised manuscript was to analyze usefulness, efficacy and safety of HFJV during stereotactic ablation of liver tumors with particular emphasis on gas exchange. The study was performed on 25 patients with malignant liver tumors. In conclusions, authors demonstrated possibility and benefits of HFJV’s usage during the liver’s surgical procedures. However, influence of some disadvantages on patients’ gas exchange was also critically discussed.

 In general the work presents interesting results and, in my opinion, could be accepted.

I confirm that I have read this submission and believe that I have an appropriate level of expertise to confirm that it is of an acceptable scientific standard.
